# Hepatic Arterial Infusion Chemotherapy for Patients with Huge Unresectable Hepatocellular Carcinoma

**DOI:** 10.1371/journal.pone.0092784

**Published:** 2014-05-13

**Authors:** Wei-Lun Tsai, Kwok-Hung Lai, Huei-Lung Liang, Ping-I Hsu, Hoi-Hung Chan, Wen-Chi Chen, Hsien-Chung Yu, Feng-Woei Tsay, Huay-Min Wang, Hung-Chih Tsai, Jin-Shiung Cheng

**Affiliations:** 1 Division of Gastroenterology, Department of Internal Medicine, Kaohsiung Veterans General Hospital, Kaohsiung, Taiwan; 2 School of Medicine, National Yang-Ming University, Taipei, Taiwan; 3 Department of Radiology, Kaohsiung Veterans General Hospital, Kaohsiung, Taiwan; 4 Department of Finance and Banking, College of Business and Management, Kun Shan University, Tainan, Taiwan; University of Modena & Reggio Emilia, Italy

## Abstract

**Background and Aim:**

The optimal treatment for huge unresectable hepatocellular carcinoma (HCC) remains controversial. The outcome of transcatheter arterial chemoembolization (TACE) for patients huge unresectable HCC is generally poor and the survival benefit of TACE in these patients is unclear. The aim of the study is to compare the effect of hepatic arterial infusion chemotherapy (HAIC) versus symptomatic treatment in patients with huge unresectable HCC.

**Methods:**

Since 2000 to 2005, patients with huge (size >8cm) unresectable HCC were enrolled. Fifty-eight patients received HAIC and 44 patients received symptomatic treatment. In the HAIC group, each patient received 2.4+1.4 (range: 1–6) courses of HAIC. Baseline characteristics and survival were compared between the HAIC and symptomatic treatment groups.

**Results:**

The HAIC group and the symptomatic treatment group were similar in baseline characteristics and tumor stages. The overall survival rates at one and two years were 29% and 14% in the HAIC group and 7% and 5% in the symptomatic treatment group, respectively. The patients in the HAIC group had significantly better overall survival than the symptomatic treatment group (P<0.001). Multivariate analysis revealed that HAIC was the significant factor associated with the overall survival (relative risk: 0.321, 95% confidence interval: 0.200–0.515, P<0.001). None of the patients died due to immediate complications of HAIC.

**Conclusions:**

HAIC is a safe procedure and provides better survival than symptomatic treatment in patients with huge unresectable HCC.

## Introduction

Hepatocellular carcinoma (HCC) is the fifth most common cancer in the world and it is the 2^nd^ leading cause of cancer death in Taiwan [1], [2]. Although high- risk patients are routinely screened in Taiwan, huge HCCs with size of more than 8 cm are occasionally seen [3]. Treating patients with huge HCC is difficult. Surgical resection is considered to be the standard curative therapy for HCC in patients with good liver reserve [4]–[8]. According to the study from our hospital, Mok et al. found that the advantage of hepatic resection in patients with huge HCC is marginal as compared with multimodality treatment including transcatheter arterial embolization (TAE), hepatic arterial infusion chemotherapy (HAIC) or local ablation therapy [9]. However, huge HCC often presented with poor liver reserve, with increased frequency of intrahepatic metastasis and vascular invasion, which made surgical resection unsuitable. So transcatheter arterial embolization/chemoembolization (TAE/TACE) has been considered as the choice for the palliative treatment of huge unresectable HCC [10]. However, previous studies found that TACE for huge HCC had poor effect, and TACE related mortality rate of 6.5–20% were reported [10]–[11]. HAIC is another option for palliative treatment for inoperable advanced HCC [12]–[15]. From our study in 2004, HAIC with cisplatin, mitomycin C, leucovorin and 5-FU for advanced unresectable HCC has a tumor response rate of 28.3% and only one patient died due to the complication of HAIC during 211 courses of treatments [16]. From another recent study of our hospital, HAIC for advanced HCC had an overall response rate of 20% [17]. However, the effect of HAIC for the treatment of huge unresectable HCC remained unclear.

The aim of this study is to investigate the effect of HAIC versus symptomatic treatment for the treatment of huge unresectable HCC.

## Materials and Methods

### Patients

From Janunary 2000 to December 2005, consecutive eligible patients with hepatocellular carcinoma (HCC) were enrolled in this study. HCC was diagnosed by pathology or elevation of alpha-fetoprotein (AFP) level above 400 ng/ml along with at least two different imaging techniques. All patients met the following criteria: (a) tumor of 8 cm or more in diameter, (b) patients who were not suitable for operation, (c) platelet counts >50000/cumm, (d) prothrombin time INR <1.5. (e) white cell counts >2500/cumm, and (f) Child A or B liver reserve. Patients with a previous history of treatment for HCC, or distant metastasis were excluded. Fifty-eight patients who received HAIC and 44 patients who received symptomatic treatment entered this study. In Taiwan, many patients did not like to receive chemotherapy, although they can fit the criteria for HAIC. Among the 44 patients who received symptomatic treatment, 16 patients had patent portal vein, but they refused TAE/TACE and another 28 patients who had thrombosis of portal vein refused HAIC.

### Ethics Statement

The study was approved by the Kaohsiung Veterans General Hospital Institutional Review Board (VGHKS13-CT11-10). This was a retrospective study without intervention or obtaining clinical specimens and all the data were analyzed anonymously, so informed consent was waived. The waiving of informed consent was approved by the Institutional Review Board of Kaohsiung Veterans General Hospital.

### Hepatic Arterial Infusion Chemotherapy (HAIC)

The left subclavian artery was cannulated with a catheter and the tip of the catheter was placed in the proper hepatic artery under fluoroscopic guidance before each course of chemotherapy [13]. The main trunk of the gastroduodenal artery was occluded by metallic coil routinely. Continuous infusion of 5000 units (5cc) heparin solution daily was filled in the catheter for preventing occlusion by thrombosis. Each course of treatment was for 5 days. Cisplatin (10 mg/m^2^) and mitomycin-C (2 mg/m^2^) were dissolved in 50 ml isotonic sodium chloride solution which was infused for 20–30 minutes each time and continued for 5 days. In addition, 100 mg/m^2^ of 5-fluorouracil (5-FU), dissolved in 250 ml of isotonic sodium chloride solution was administered for 24 hours using an infusion pump for 5 days. Leucovorin (15 mg/m^2^) was given daily to improve the efficacy of 5-FU during HAIC. The interval between 2 courses of treatment was 3 to 4 weeks. Each patient received at least one session of treatment. Three-phase computed tomography (CT) scan of liver was done after every 2 courses of treatment. The treatment was terminated when patients received 6 courses of treatment or until clinical conditions of the patients were not suitable for another course of HAIC.

### Follow-up

All patients in the symptomatic treatment group and in the HAIC group who completed chemotherapy received follow-up with liver function test, AFP, sonography and/or CT scan every 3–6 months.

### Assessment of Response

The modified Response Evaluation Criteria in Solid Tumors (RECIST) was used for assessment of tumor response defined as follows: complete response: no evidence of neoplastic disease; partial response: At least a 30% decrease in the sum of diameters of viable (enhancement in the arterial phase) target lesions, taking as reference the baseline sum of the diameters of target lesions; stable disease: any cases that do not qualify for either partial response or progressive disease; progressive disease: an increase of at least 20% in the sum of the diameters of viable (enhancing) target lesions, taking as reference the smallest sum of the diameters of viable (enhancing) target lesions recorded since treatment started.

### Statistical Analysis

The data were expressed as mean+standard deviation. Categorical variables were compared with the *X* ^2^ test or Fisher’s exact test when appropriate and continuous variables were compared with the Mann-Whitney test. Overall survival was estimated using the Kaplan –Meier method and the difference was determined by the log-rank test. Univariate and multivariate analysis with age (>65 vs <65 years), sex (male vs. female), vascular invasion, Child classification (A vs B), Okuda stage (I vs. II–III), Cancer of the Liver Italian Program (CLIP) score (1–3 vs. 4–6), Barcelona Clinic Liver Cancer (BCLC) stage (A–B vs. C–D), AFP level (>1000 vs <1000 ng/ml), and treatment (HAIC vs. symptomatic treatment) were performed using Cox’s regression model with proportional hazards. A P-value of less than 0.05 was considered as statistically significant.

## Results

The baseline characteristics of patients in the HAIC group and the symptomatic treatment group were similar in age, sex, tumor size, tumor number, the presence of main portal vein invasion, albumin level, bilirubin level, Child classification, Okuda stage, CLIP score, and BCLC stage (table 1).

**Table 1:** Baseline characteristics of the patients.

	HAIC N = 58	Symptomatic treatment N = 44	P-value
Age (years) a	61±12	63±17	.407
Sex (M/F)	50/8	32/12	.130
HBV/non-HBV	35/23	20/24	.135
Tumor size (cm) a	11±3	11±3	.277
Tumor No (1/>1)	25/33	18/26	.824
Albumin (g/dl) a	3.3±0.6	3.1±0.5	.177
Bilirubin (mg/dl) a	1.3±0.7	1.5±0.8	.262
AFP(>1000/<1000 ng/ml)	31/27	24/20	1.000
Main portal vein invasion (+/−)	32/26	28/16	.390
Child class (A/B)	30/28	20/24	.530
Okuda stage(1/2–3)	6/52	5/39	1.000
CLIP score(1–3/4–6)	37/21	23/21	.242
BCLC stage (A–B/C–D)	16/42	13/31	1.000

a Data presented as mean +/− standard deviation.

AFP: alpha-fetoprotein, CLIP: Cancer of the Liver Italian Program, BCLC: Barcelona-Clinic Liver Cancer.

A total of 134 courses of HAIC were performed for the 58 patients in the HAIC group. Each patient received 2.4+1.4 (range: 1–6) courses of HAIC. None of the patients died due to immediate HAIC complications. Adverse events of patients who received HAIC according to National Cancer Institute Common Terminology Criteria for Adverse Events (NCI-CTC AE) grading were described in Table 3. Eight patients developed fever during HAIC and among them, four patients developed bacteremia and were treated successfully by antibiotics. One patient developed overt subcutaneous hematoma at the puncture site but recovered soon and did not require further management. No vascular complications including occlusion or vasculitis of the hepatic artery were found.

**Table 3:** Adverse events of patients who received HAIC according to NCI-CTC AE grading.

Clinical toxicities	Grade I–II, n (%)	Grade III–IV, n (%)
Leukopenia	7 (12)	2 (3.4)
Anemia	3 (5.2)	0 (0)
Thrombocytopenia	4 (6.9)	0 (0)
Elevated aminotransferase	8 (13.8)	3 (5)
Hyperbilirubinemia	8 (13.8)	0 (0)
Elevated creatinine	2 (3.4)	0 (0)
Nausae/vomiting	25 (43)	0 (0)
Diarrhea	5 (8.6)	0 (0)
Fever	4 (6.9)	4 (6.9)

**Figure 1:**
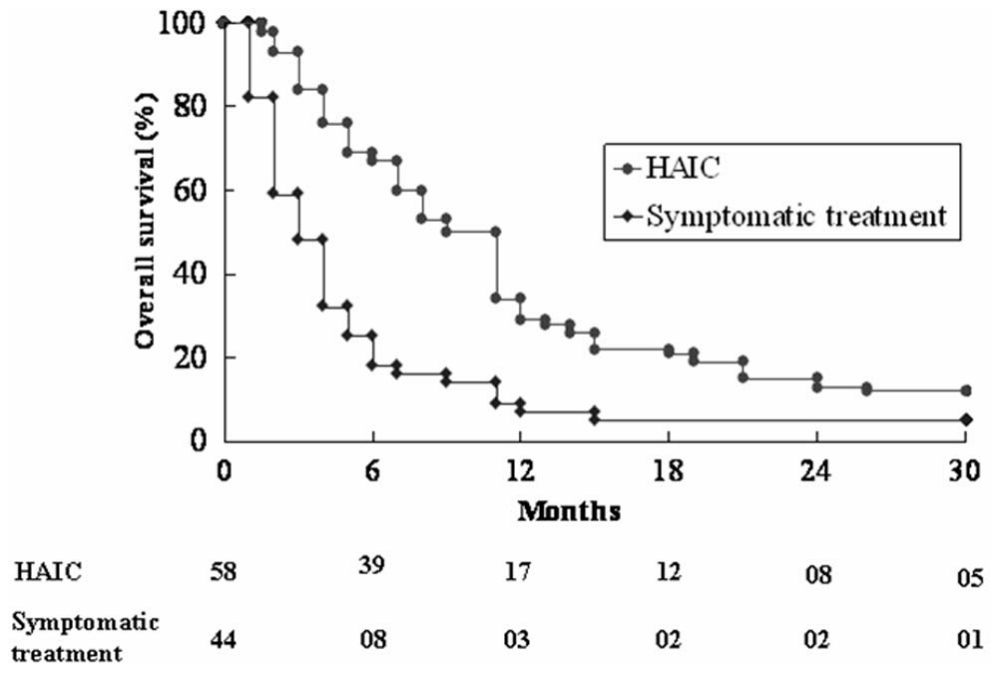
Comparison of the overall survival rate between the HAIC and symptomatic treatment group. The HAIC group had significantly better survival than the symptomatic treatment group (P<0.001).

Mean follow-up time was 10+11 months (range: 1–52 months). The median survival time is 9.5 (1.5–50) months in the HAIC group and 3.0 (1–45) months in the symptomatic treatment group (P = 0.001). The overall survival rates at one and two years were 29% and 14% in the HAIC group and 7% and 5% in the symptomatic treatment group. The patients in the HAIC group had significantly better overall survival than those in the symptomatic treatment group (P<0.001) (Figure 1). Univariate and multivariate analysis of factors associated with mortality was shown in Table 2. Multivariate analysis revealed that the significant factors associated with the overall survival were treatment method (HAIC vs. symptomatic treatment, relative risk (RR): 0.321, 95% confidence interval (CI): 0.200–0.515, P<0.001), Child classification (A vs. B, RR: 0.537, 95% CI: 0.321–0.901, P = 0.019) and CLIP score (1–3 vs 4–6, RR:0.611, 95% CI: 0.375–0.997, P = 0.048) (table 2).

**Table 2:** Factors associated with overall mortality in patients who receive HAIC or symptomatic treatment.

	Univariate analysis			Multivariate analysis		
	Hazard ratio	95% CI	P	Hazard ratio	95% CI	P
Age (>65 vs. <65 yrs)	**1.175**	**0.774–1.785**	**0.449**			
Sex (Male vs. female)	**1.113**	**0.655–1.890**	**0.692**			
Child’s classification (A vs. B)	**0.430**	**0.279–0.662**	**<0.001**	**0.537**	**0.321–0.901**	**0.019**
PV thrombosis (yes vs. no)	**1.346**	**0.880–2.059**	**0.171**			
AFP level (>1000 vs <1000 ng/ml)	**0.683**	**0.450–1.036**	**0.073**			
Okuda stage (I vs. II–III)	**0.608**	**0.311–1.189**	**0.146**			
HAIC vs symptomatic treatment	**0.394**	**0.258–0.603**	**<0.001**	**0.321**	**0.200–0.515**	**<0.001**
CLIP sore (1–3 vs. 4–6)	**0.482**	**0.315–0.736**	**0.001**	**0.611**	**0.375–0.997**	**0.048**
BCLC stage (A–B vs. C–D)	**0.562**	**0.348–0.908**	**0.018**			

HAIC: Hepatic arterial infusion chemotherapy, CLIP: Cancer of the Liver Italian Program, BCLC: Barcelona Clinic Liver Cancer.

Tumor responses could be evaluated in 53 patients (39 in the HAIC group and 14 patients in the symptomatic treatment group) who survived for more than 3 months and had a measurable tumor on CT scan. In the HAIC group, 7 had responses (2 had complete responses and 5 had partial responses), 12 had stable diseases and 20 had progressive diseases. In the symptomatic treatment group, no patients had responses, 5 had stable diseases and 9 had progressive diseases. The rate of tumor response in measurable patients was higher in the HAIC group than the symptomatic treatment group but not statistically significant (P = 0.176).

## Discussion

Huge HCC is difficult to treat. Surgical resection is the treatment of choice for patients with huge HCC and well-preserved liver function [7], [18]. However, only a small proportion of patients with huge HCC can fit the criteria for surgical resection. But patients with huge HCC often had a higher prevalence of extracapsular tumor invasion into liver parenchyma, more frequent intrahepatic metastasis and worse survival than those with smaller tumors [19]–[21]. There remained some controversies regarding the treatment for huge unresectable HCC.

During the 134 courses of HAIC, most patients tolerated the procedure well and no patients died of the immediate complications of HAIC. So HAIC may be a more safe treatment option for huge HCC.

The overall response rate in the HAIC group was 18% but no patients in the symptomatic treatment group had response. Response was evaluated only in patients who survived for more than 3 months. Twenty-one out of 44 patients in the symptomatic treatment group survived for more than 3 months. The better survival in the HAIC group may be due to the higher response rate.

Patients with huge unresectable HCC were hard to treat and had poor prognosis. Two-year survival rate for patients with huge unresectable HCC who received symptomatic treatment was only 5% in this study. This finding was in accordance with the result of previous study by Huang et al [10], who found that the two-year survival rate for patients with huge unresectable HCC who received symptomatic treatment was 7.7%. This study found that HAIC had survival benefit over symptomatic treatment for huge unresectable HCC, and was also a safe treatment procedure. Therefore, we should encourage patients with huge HCC to receive HAIC to prolong survival if surgical resection was not suitable.

According to the study by Yamasaki et al [12], tumor size was not a prognostic factor that influenced the outcome of HAIC for patients with advanced HCC. But large tumor size was found to be associated with poor outcome of TACE for patients with HCC [11], [22], [23]. Although TAE/TACE has been considered as the palliative treatment for huge unresectable HCC in many institutes, the treatment outcome is generally poor [10]–[11], the survival benefit of TAE/TACE in these patients remained controversial and the mortality rate due to immediate complications of TAE/TACE for huge HCC ranged from 6.5–20% in previous studies [10], [11]. In our unpublished data that compared the outcome of HAIC and TAE for huge HCC, we found that the overall survival rates of the HAIC group was higher than the TAE group but did not reach statistical significance (P = 0.077). Besides, 12% of patients in the TAE group died due to the immediate complications. In this study we found HAIC is safe and had survival benefit over symptomatic treatment for huge unresectable HCC. Further randomized controlled study to compare the treatment outcome of HAIC versus TAE/TACE for huge unresectable HCC is required. Besides, main portal vein thrombosis was found in 60 out of 102 patients with huge unresectable HCC in this study. Main portal vein thrombosis was a contraindication for TAE/TACE but not a contraindication for HAIC. So HAIC should be considered as an important treatment option for huge unresectable HCC especially when main portal vein thrombosis was present.

Multivariate analysis found that HAIC, Child classification and CLIP score were significantly associated with better survival rate. Child classification has been found to be associated with the outcome of patients with HCC treated with HAIC, TACE [12]–[13] or local ablation therapy [24] in our study. CLIP score also was found to be associated with the outcome of patients with HCC treated with TACE [25]–[26]. Both liver reserve and tumor stage remained to be the important prognostic factors in patients with huge unresectable HCC.

Sorafenib has been developed and is recommended for the treatment of advanced HCC [27], [28]. But the effect of sorafanib for HCC is not satisfactory and actually the response rate of sorafenib is low. In the Asia-Pacific study by Cheng et al, the overall survival was 6.5 months in patients treated with sorafenib, compared with 4.2 months in those who received placebo and only 3.3% of patients achieved a partial response and no patients had complete response [29]. Effects of sorafenib in patients with huge unresectable HCC is unclear. Besides, sorafenib is limited by a high cost and many patients can not afford to receive the treatment. The treatment outcome of TACE for patients with huge unresectable HCC is generally poor [10]–[11]. In countries where sorafenib is not available or too expensive for patients to afford, HAIC is a good treatment option.

HAIC has been found to have benefits in HCC patients with portal vein thrombosis (PVT) [30], [31]. Recently Lau et al reported that transarterial radioembolization (TARE) is a good treatment option in HCC patients with PVT [32]. In patients with unresectable HCC who have developed PVT, TARE may prolong survival with minimal impact on quality of life. Further studies are required to compare the effects of HAIC vs. TARE in patients of HCC with PVT.

This study has several limitations. This is not a randomized controlled study, and selection bias may be possible in this study. But the baseline characteristics including age, sex, liver reserve, tumor stages are similar between the two groups of patients and after multivariate analysis, the HAIC group still has survival benefit over patients who received symptomatic treatment. In a recent non-randomized study from Japan [30], Nouso et al. found HAIC has survival benefit over symptomatic treatment in advanced HCC. This is in accordance with the result of our study. A randomized controlled study to compare the treatment outcome of HAIC versus symptomatic treatment for huge unresectable HCC has not been reported before and it is not ethical to place patients untreated during randomization.

In conclusion, HAIC is a safe procedure and provides better survival than symptomatic treatment for patients with huge unresectable HCC.
